# Computed Tomography Radiomics for Predicting Pathological Grade of Renal Cell Carcinoma

**DOI:** 10.3389/fonc.2020.570396

**Published:** 2021-01-27

**Authors:** Xiaoping Yi, Qiao Xiao, Feiyue Zeng, Hongling Yin, Zan Li, Cheng Qian, Cikui Wang, Guangwu Lei, Qingsong Xu, Chuanquan Li, Minghao Li, Guanghui Gong, Chishing Zee, Xiao Guan, Longfei Liu, Bihong T. Chen

**Affiliations:** ^1^ Department of Radiology, Xiangya Hospital, Central South University, Changsha, China; ^2^ Department of Urology, Xiangya Hospital, Central South University, Changsha, China; ^3^ Department of Pathology, Xiangya Hospital, Central South University, Changsha, China; ^4^ Xiangya School of Medicine, Central-South University, Changsha, China; ^5^ School of Mathematics and Statistics, Central South University, Changsha, China; ^6^ Department of Radiology, Keck School of Medicine, University of Southern California, Los Angeles, CA, United States; ^7^ Department of Diagnostic Radiology, City of Hope National Medical Center, Duarte, CA, United States

**Keywords:** radiomics, clear cell renal cell carcinoma, computed tomography (CT), machine learning, predictive modeling

## Abstract

**Background:**

Clear cell renal cell carcinoma (ccRCC) is the most common renal cancer and it has the worst prognosis among all renal cancers. However, traditional radiological characteristics on computed tomography (CT) scans of ccRCC have been insufficient to predict the pathological grade of ccRCC before surgery.

**Methods:**

Patients with ccRCC were retrospectively enrolled into this study and were separated into two groups according to the World Health Organization (WHO)/International Society of Urological Pathology (ISUP) grading system, i.e., low-grade (Grade I and II) group and high-grade (Grade III and IV) group. Traditional CT radiological characteristics such as tumor size, pre- and post-enhancing CT densities were assessed. In addition, radiomic texture analysis based on the CT imaging of the ccRCC were also performed. A CT-based machine learning method combining the traditional radiological characteristics and radiomic features was used in the predictive modeling for differentiating the low-grade from the high-grade ccRCC. Model performance was evaluated with the receiver operating characteristic curve (ROC) analysis.

**Results:**

A total of 264 patients with pathologically confirmed ccRCC were included in this study. In this cohort, 206 patients had the low-grade tumors and 58 had the high-grade tumors. The model built with traditional radiological characteristics achieved an area under the curve (AUC) of 0.9175 (95% CI: 0.8765–0.9585) and 0.8088 (95% CI: 0.7064–0.9113) in differentiating the low-grade from the high-grade ccRCC for the training cohort and the validation cohort respectively. The model built with the radiomic textural features yielded an AUC value of 0.8170 (95% CI: 0.7353–0.8987) and 0.8017 (95% CI: 0.6878–0.9157) for the training cohort and the validation cohort, respectively. The combined model integrating both the traditional radiological characteristics and the radiomic textural features achieved the highest efficacy, with an AUC of 0.9235 (95% CI: 0.8646–0.9824) and an AUC of 0.9099 (95% CI: 0.8324–0.9873) for the training cohort and validation cohort, respectively.

**Conclusion:**

We developed a machine learning radiomic model achieving a satisfying performance in differentiating the low-grade from the high-grade ccRCC. Our study presented a potentially useful non-invasive imaging-focused method to predict the pathological grade of renal cancers prior to surgery.

## Introduction

Clear cell renal cell carcinoma (ccRCC) constitutes 70%–80% of all renal cancers (1–3) and it has a poor prognosis with a cure rate under 70% even for a localized ccRCC treated by radical nephrectomy (4–6). A novel four-tiered World Health Organization (WHO)/International Society of Urological Pathology (ISUP) grading (7) has been reported to have the potential to predict prognosis in patients with ccRCC (8–10) who may have the poorest prognosis among all patients with renal cancer (11–13). The ccRCC tumors are usually subclassified into two groups including the low-grade (Grade I and II) and the high-grade (Grade III and IV) groups, reflecting the significant difference in treatment strategy and prognosis between the two groups (7, 10, 14). It has been shown that the higher ISUP grade of ccRCC has greater biologic aggressiveness, and is associated with worse survival (8, 9) and higher risk for recurrence after partial (nephron-sparing) nephrectomy (15). Knowledge of ISUP grade prior to surgery could guide clinical decision making (5, 16, 17). In addition, reliable ISUP grade obtained from a non-invasive method such as imaging may alleviate the need for renal biopsy (18), thus avoiding the risk of complications from invasive biopsies such as bleeding, infection, tumor seeding the biopsy needle path, and the relatively low accuracy in assessing tumor grade based on the biopsy specimen (19). Therefore, there is an unmet need to develop non-invasive methods for assessing the pathological grade of ccRCC before surgery.

Non-invasive imaging-based method has been used in assessing pathological grade of ccRCC before surgery (20–22). Several traditional radiological characteristics such as tumor size and CT enhancement patterns have been shown to be correlated with the tumor grade (23). However, it has been challenging to predict the pathological grade of ccRCC with the existing limited information obtained from the traditional radiological characteristics (21, 24). By contrast, radiomic analysis involving the computerized extraction of data not discernable to the human eyes could generate highly detailed imaging features regarding tumor texture, shape, and image intensity (25, 26). Such methods have been successfully used in cancer research (25, 26), presenting the potential for identifying tumor phenotype, pathological grade (27), and biological behavior (28). Therefore, radiomic analysis is a potentially useful method that could be used not only to evaluate tumor heterogeneity but also to assess pathological grade for guiding personalized cancer treatment. However, there has been limited progress in developing non-invasive radiomic machine-learning models to accurately differentiate the low-grade from the high-grade ccRCC.

In this study, we analyzed the traditional radiological characteristics of ccRCC on pre-surgical CT images including the tumor size and CT density values. In addition, we performed predictive modeling combining the features obtained from both the traditional radiological assessment and the radiomic textural analysis. We aimed to develop a radiomic machine learning model to predict the ISUP grade of ccRCC tumors pre-surgically. We hypothesized that integration of radiomic features into the traditional radiological characteristics should improve the model performance in differentiating the low-grade from the high-grade ccRCC than using either the radiomic features or the traditional radiological characteristics alone in building the model.

## Methods

### Patients

**Figure 1:**
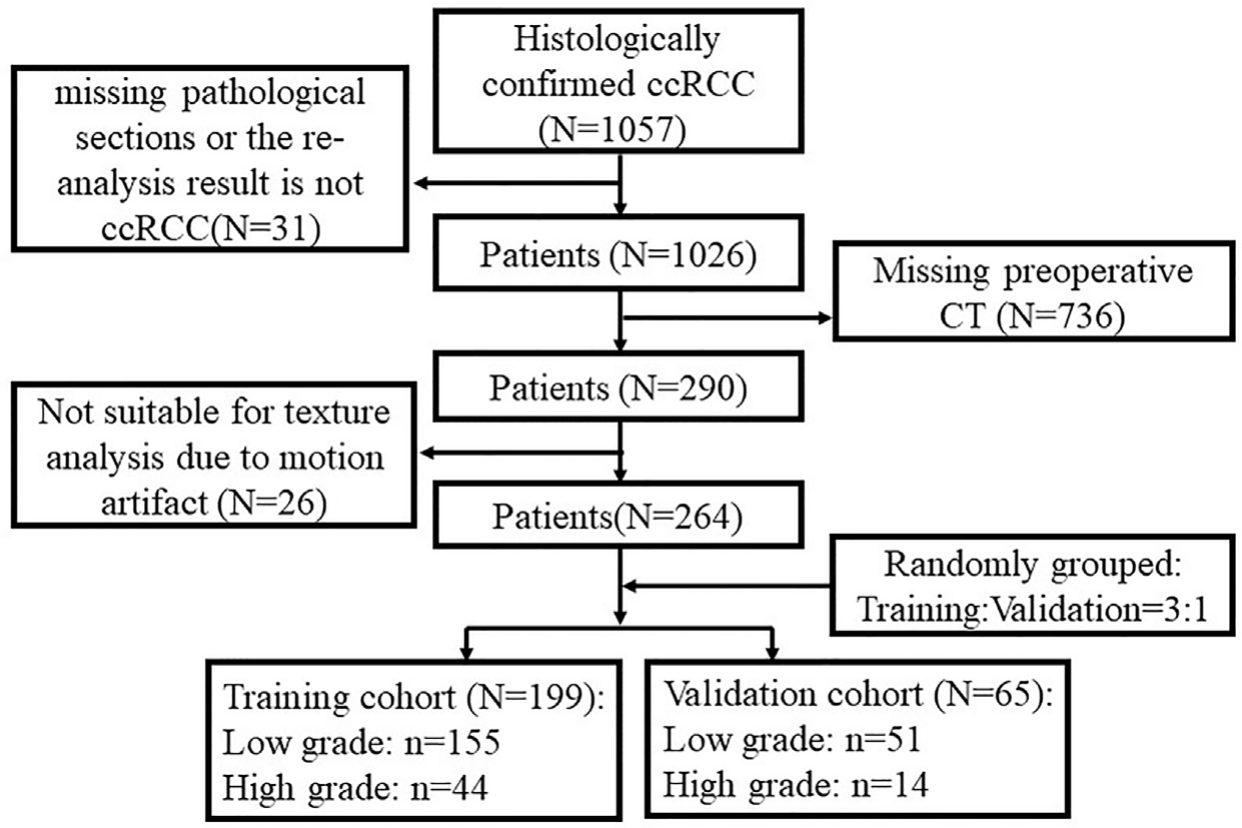
Study recruitment diagram with respect to inclusion and exclusion criteria.

Patients were consecutively identified and retrospectively included into this study through a careful assessment of our medical records from June 1, 2010 to June 1, 2017. All patients in this cohort underwent radical or partial nephrectomy with curative intent in our hospital with a final pathological diagnosis of ccRCC. Those patients with complete medical records including pathological confirmation and pre-surgical CT images were included, and their medical data including CT images was collected by research personnel (QX, FZ, ZL, and CW) for subsequent assessment. To avoid possible observer bias, the researchers were tasked specifically for different aspects of the study. For example, researcher 1 (ZL) with exposure to the original data completed the data anonymization procedure and did not participate in the subsequent analysis. The reminder three researchers including FZ only dealt with anonymized data and they were blinded to all radiological and clinicopathological information of the patients. All enrolled patients were divided into two cohorts, i.e., the training cohort and the validation cohort, at a ratio of 3:1 randomly. Details of the exclusion criteria and the patient recruiting process were shown in **Figure 1**.

This study was approved by Ethic Committee and Institutional Review Board in Xiangya Hospital of Central South University, P. R. China (IRB#2017121011). Written informed consents were waived due to the retrospective nature of this study.

### Re-Analysis of Pathological Slides

For each patient, all pathological slides (including hematoxylin and eosin [HE] and immunohistochemical [IHC] staining) were re-analyzed by two pathologists specialized in urology (GG and HY, with 6 and 25 years of experience in uropathology, respectively). Each ccRCC grading was undertaken according to the criteria of the ISUP grading system (7) (**Supplementary Table 1**), and the ccRCC tumors were separated into two groups: the low-grade group (Group 1: Grade I and II) and the high-grade group (Group 2: Grade III and IV) (14). Consensus was reached by discussion if differences in opinions existed.

### Computed Tomography Imaging

All patients had a routine abdominal CT scan obtained on one of our three CT scanners, i.e., a 16-MDCT (Brilliance 16, Philipps), a 64-MDCT (SOMATOM Definition, Siemens), or a 320-MDCT (Aquilion ONE, Toshiba Medical Systems) scanner. CT imaging included an acquisition of a pre-contrast phase and a contrast-enhanced phase with a power injector (Ulrich CT plus 150, Ulrich Medical, Ulm, Germany). Briefly, 90–100 ml of iodinated contrast material (Ultravist 370, Bayer Schering Pharma, Berlin, Germany) was administered intravenously at a rate of 3.0–3.5 ml per second. Contrast-enhanced images at the nephrographic phase (scan with fixed delay time of 65 s) were obtained for all patients. Since all patients had CT images for both the non-enhanced phase and the nephrographic/portal venous phase, the CT images from these two phases were included in this analysis. All CT images were retrieved from our Picture Archiving and Communication Systems (PACS, Carestrem, Canada), and were downloaded to an external workstation (Leonardo; Siemens Medical Solutions, Forchheim, Germany). All CT images were reconstructed into the voxel size of 1×1×1mm^3^ for subsequent analysis.

### Traditional Radiological Analysis

Two radiologists specialized in abdominal imaging (Reader 1: FZ with 10 years of experience and Reader 2: GL with 25 years of experience) reviewed the CT images independently. They were blinded to all radiological and clinicopathological information of the patients. They recorded the traditional CT imaging findings including the tumor size measurements such as transverse dimension in millimeter (mm), anteroposterior dimension (mm), cranio-caudal dimension (mm), pre-enhanced CT density value (CTpre) in Hounsfield units (HU), enhanced CT density value (CTpost) in HU, and enhancement range in HU.

### Radiomic Textural Feature Extraction

We used the pre-contrast non-enhanced CT images for radiomic textural feature extraction due to the following reasons. In this retrospective study with the images already acquired, we were concerned about the potential confounding variables affecting the contrast-enhanced images such as the inconsistent injection speed of contrast medium and varying hemodynamics of each patient after contrast administration. These variables may contribute to varying contrast enhancement of the tumors that did not reflect the true tumor heterogeneity. On the contrary, pre-contrast non-enhanced CT images were easy to acquire and were relatively stable from one patient to another, which may show the inherent tumor heterogeneity. In our study, the radiomic textural feature extraction was performed only on the non-enhanced images.

For each patient’s CT scan, a representative axial image with the largest cross-sectional measurement of the renal tumor was selected. In order to eliminate the potential variance of CT images obtained on the three different scanners, all original CT images underwent normalization using the gray-scale discretization method before textural feature extraction, with a final 256 bins (Analysis Kit software, version V3.0.0.R, GE Healthcare) (29, 30). Subsequently, we used the textural analysis software (MaZda Version 4.6, Institute of Electronics, Technical University of Lodz, Poland) (31) to perform the image analysis. A region of interest (ROI) to outline the tumor boundaries was drawn manually. The corresponding contrast-enhanced CT images were used as references in delineating the precise boundaries of the tumor on pre-enhanced images. All contouring was reviewed and validated by two senior abdominal radiologists (XY and GL) with 15 and 16 years of experience, respectively, in interpreting genitourinary CT images.

For each patient, a total of 340 textural features were extracted with the MaZda software based on corresponding ROI file, including a gray-level histogram, a gradient, a run-length matrix, a co-occurrence matrix, an autoregressive model and a wavelet transform analysis.

### Reproducibility of Textural Feature Extraction

To evaluate the reproducibility of the radiomic textural feature extraction, the inter-observer (Reader 1 versus Reader 2) and intra-observer (Reader 1 twice) correlation coefficient (ICC) values were accessed. The reader consistency and reproducibility were determined according to the following criteria based on the ICC value: poor (<0.20), fair (0.21–0.40), moderate (0.40–0.60), good (0.61–0.80), and excellent (0.81–1.00). In general, an ICC exceeding 0.75 indicated good agreement.

The differences of the values for each feature between the two groups, and the differences between the textural features generated by Reader 1 (first time) and those by Reader 2, as well as between the features twice-generated by Reader 1, were analyzed using Mann-Whitney U test, independent samples t-test or Kruskal-Wallis H test, where appropriate.

Inter-observer and intra-observer reproducibility was initially analyzed with 50 randomly chosen patients’ CT images evaluated by two radiologists (Reader 1 and Reader 2). To assess the inter-observer reproducibility, Reader 1 and Reader 2 completed the workflow as described previously (32).

A 0.2–1 cm^2^ circular ROI was used to measure CT attenuation values of the tumors in HU. ROIs were placed on the solid parts of the tumor for three times, then the average CT attenuation value was recorded. Tumor size measurements including transverse dimension (mm), anteroposterior dimension (mm), and cranio-caudal dimension (mm), were all measured three times, and then the average values were recorded.

### Statistical Analysis, Feature Selection, and Prediction Model Building

IBM SPSS version 22.0.0 (IBM Corporation, Armonk, NY, USA) was used to for statistical analyses. The differences about quantitative radiomic features and the qualitative features between the two groups, i.e., the low-grade group and the high-grade group, were tested using the Wilcoxon rank-sum test and the chi-square test respectively.

We used MATLAB 2017a (The Mathworks, Inc., Natick, MA, USA) to perform the data processing, data reduction for feature selection, and model building. The least absolute shrinkage and selection operator (LASSO) method was performed to select the features from the training cohort that possessed the most useful predictive value. Based on these selected features, machine learning methods including the Random Forest (RF) method and the support vector machine (SVM) method were used to generate the differentiation models according to the classification algorithm developed in our previous report (32).

**Figure 2:**
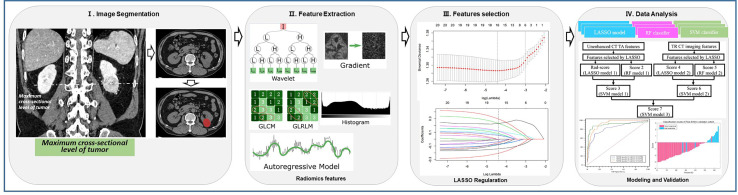
Flow chart for radiomic feature extraction, feature selection and predictive modeling. (I) Representative tumor segmentation computed tomography (CT) images. (II) Radiomic feature extraction. (III) Radiomic feature selection. (IV) Classification algorithm and predictive modeling.

The differentiation models were developed in the training cohort, and were validated in the validation cohort. The classification efficiencies of the models were calculated using the receiver operating characteristic (ROC) curves analysis. A P value < 0.05 was considered statistically significant. The work flow for radiomic feature extraction, feature selection and classification model building was presented in **Figure 2**. Details of the flow chart depicting the process of predictive modeling was shown in **Supplementary Figure 1**.

### Correlation Test Among Selected Features

A correlation matrix analysis was performed to evaluate associations between the radiomic textural features and the traditional radiological characteristics, including correlations of features within each of these two groups, i.e., the low-grade group and the high-grade group as well as between the two groups.

## Results

### Patient Characteristics Between the Training and Validation Cohorts

**Table 1** summarized the clinicopathological and traditional radiological characteristics of this study cohort. There were no significant differences in the clinical characteristics such as gender and age between the training and the validation cohorts. There were no significant differences in the distribution of the low-grade and the high-grade ccRCC between the two cohorts. No significant differences were noted between the two cohorts regarding the tumor size measurements or CT density values.

**Table 1:** Comparison of patient characteristics between the training cohort and the validation cohort.

	Training cohort	Validation cohort	P value
Gender			0.923
Male	141(70.85%)	42(64.62%)	
Female	58(29.15%)	23(35.38%)	
Age (years)	53.22 ± 11.05	54.09 ± 10.78)	0.789
Group			0.344
Low ISUP grade	155 (77.89%)	51 (78.46%)	
High ISUP grade	44 (22.11%)	14 (21.54%)	
T-stage			0.817
T1	110 (55.28%)	37 (56.92%)	
T2	89 (44.72)	28 (43.08%)	
Tumor size (mm)			
transverse	4.46 (1.36–11.55)	4.49 (1.78–8.88)	0.752
anterior-posterior	4.65 (0.88–12.36)	4.56 (2.09–10.53)	0.864
cranio-caudal	4.55 (1.13–18.74)	4.76 (1.94–13.14)	0.914
CT-pre (HU)	32.18 ± 8.32	33.05 ± 6.60	0.439
CT-V(HU)	79.46 (31.69–249.91)	77.79 (31.84–152.79)	0.949
Enhancement (HU)	47.83 (9.39–221.28)	44.99 (11.40–117.40)	0.841

ISUP, International Society of Urological Pathology; CT-pre, CT value on pre-enhanced CT image; CT-V, CT value on enhanced CT image during nephrographic/portal venous phase; Enhanced, the HU values during the CT-V phase. HU, Hounsfield Units.

### Patient Characteristics Between the Low-Grade and the High-Grade Groups

The tumors size measurements in the low-grade group (Group 1) were significantly smaller than those in Group 2. The CTpre values of the Group 1 tumors were significantly lower than those of the Group 2 tumors. In contrast, tumors in Group 1 showed a higher CTpost value than the tumors in Group 2, although the difference did not reach statistical significance (*P*=0.052). However, when considering the CTpre values, the degree of enhancement for Group 1 tumors was significantly higher than that of the Group 2 tumors (*P*=0.001). Details of the corresponding statistical results were presented in **Table 2**.

**Table 2:** Comparison of patient characteristics between the low-grade and the high-grade tumors.

	Low ISUP grade	High ISUP grade	P value
Gender			0.368
Male	140(67.96%)	43(74.14%)	
Female	66(32.04%)	15(25.86%)	
Age (years)	53.19 ± 11.30	54.28 ± 9.77	0.508
T-stage			<0.001
T1	127 (61.65%)	20 (34.48%)	
T2	79 (38.35%)	38 (65.52%)	
Tumor size (mm)			
transverse	4.12 (1.36–11.55)	5.98 (1.87–10.44)	<0.001
anterior-posterior	4.32 (0.88–11.05)	6.02 (1.54–12.36)	<0.001
cranio-caudal	4.23 (1.13–12.23)	6.30 (1.91–18.74)	<0.001
CT-pre (HU)	31.26 ± 7.93	36.41 ± 6.51	<0.001
CT-V (HU)	82.17 (31.69–249.91)	73.52 (38.58–152.79)	0.052
Enhancement (HU)	49.66 (9.39–221.28)	38.76 (10.58–117.40)	0.001

ISUP, International Society of Urological Pathology; The interval values in parentheses refer minimum- maximum; CT-pre, CT value on pre-enhanced CT image; CT-V, CT value on enhanced CT image during nephrographic/portal venous phase; Enhanced, the HU values during the CT-V phase. HU, Hounsfield Units.

### Reproducibility of Radiomic Feature Extraction and Traditional Radiological Assessment

Our results demonstrated satisfactory inter- and intra-observer reproducibility of the radiomic feature extraction and the traditional radiological assessment. The inter-observer ICCs of for radiomic features between Reader 1 (first time) and Reader 2 ranged from 0.761 to 0.893. The intra-observer ICC of Reader 1 with two extraction performances ranged from 0.781 to 0.909. As a result, the radiomic features extracted by Reader 1 were used in all subsequent analysis. The inter-reader analysis achieved good to excellent agreement in traditional radiological evaluation (ICC = 0.687–0.936). The ICC values for the traditional radiological features were not high, which could be explained by the following reasons. First, the traditional radiological features such as CT density may vary from one scan to another due to inherent tumor heterogeneity. In addition, the solid components of tumors might not be homogenously enhancing and therefore may result in variations in local delineation of ROIs for CT density measurements. Second, there may be subtle differences in CT density among the three different CT scanners. Third, the renal tumors were generally small and the solid enhancing parts of the renal tumor were even smaller in size. Any minor variations in local delineation of ROI between the readers may result in a large difference in ICC. However, caution was taken in delineation of ROIs and all measurements were performed three times with the average values being recorded.

### Model Built With Radiomic Textural Features

**Figure 3:**
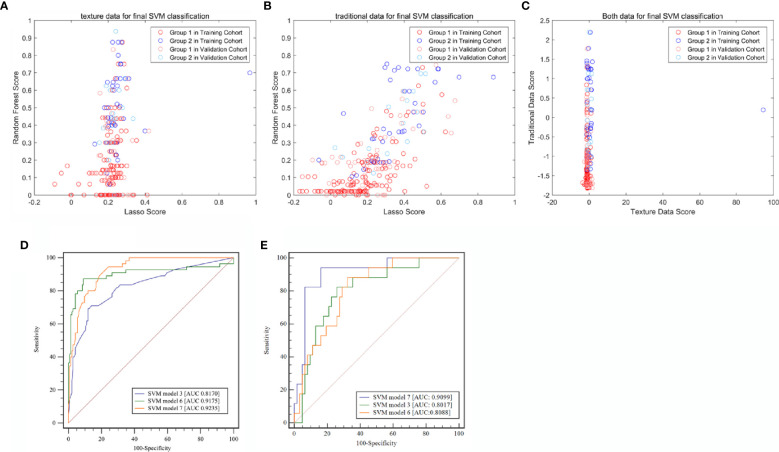
Classification efficiencies of the three support vector machine (SVM) models. **(A)** Model built with radiomic textural features. **(B)** Model built with traditional radiological characteristics. **(C)** Model built with both radiomic textural features and traditional radiological features. **(D)** Receiver operating characteristic (ROC) curve analysis for the training cohort. **(E)** Receiver operating characteristic (ROC) curve analysis for the validation cohort. LASSO, least absolute shrinkage and selection operator; SVM, Support vector machine.

A total of 340 features were extracted from pre-enhanced CT images for each patient. Of all the textural features, 19 features were finally selected to build a textural signature (Rad-score) after performing LASSO for feature selection. This process was included in the **Supplementary Files** (**Supplementary Files: Equation 1**). The same set of features was also used to build a RF classifier (score 2). A SVM classifier (SMV 1) was built based on the two models. The SVM 1 classifier achieved a classification performance with an AUC value of 0.8170 (95% CI: 0.7353–0.8987) and 0.8017 (95% CI: 0.6878–0.9157) in the training and validation cohorts, respectively (**Figure 3**).

### Model Built With Traditional Radiological Characteristics

The traditional radiological characteristics including the transverse dimension, cranio-caudal dimension, CTpre, and enhancement range were selected using the LASSO method (**Supplementary Files: Equation 2**) (score 4), and a RF model (Score 5) was built through the same modeling process as performed for the radiomic textural features. Based on scores 4 and 5, a new SVM classifier (SVM 2; Score 6) was created. The AUC of SVM2 was 0.9175 (95% CI: 0.8765–0.9585) and 0.8088 (95% CI: 0.7064–0.9113) in the training and validation cohorts, respectively.

**Figure 4:**
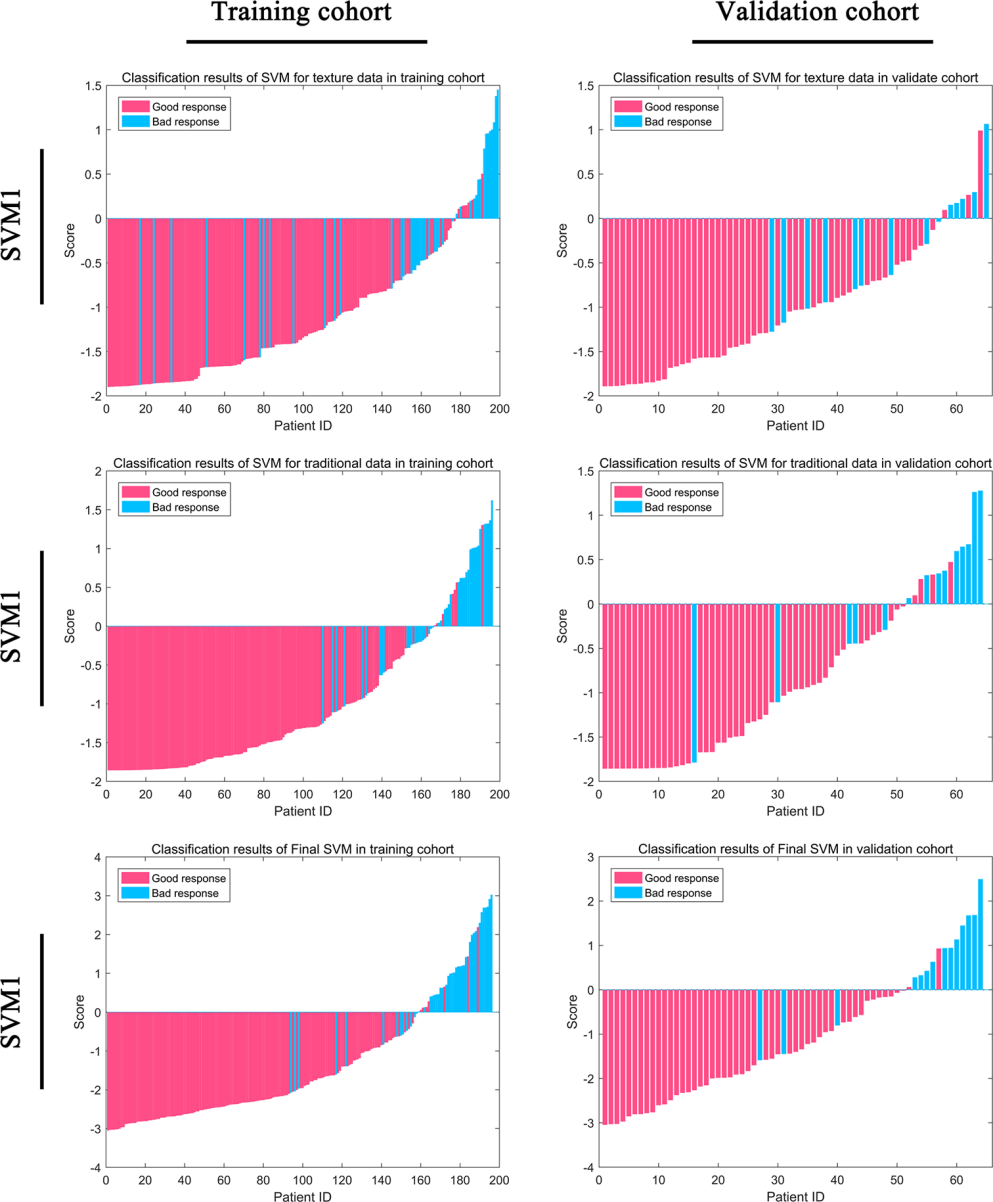
Classification efficiency for the training cohort and the validation cohort for the support vector machine (SVM) models.

Based on SVM1 (Score 3) and SVM2 (Score 6), the final classification model built by the SVM method (SVM3; Score 7) was constructed. This model provided an AUC of 0.9235 (95% CI: 0.8646–0.9824) with a sensitivity of 0.8780 (95% CI: 0.7561–0.9756) and a specificity of 0.9167 (95% CI: 0.8611–0.9722) in the training cohort, and a AUC of 0.9099 (95% CI: 0.8324–0.9873) with a sensitivity of 0.9412 (95% CI: 0.7647–1.0000) and a specificity of 0.8871 (95% CI: 0.7742–0.9839) in the validation cohort (**Table 3** and **Figure 4**).

**Table 3:** Summary of three machine learning models for predicting pathological grade based on radiomic features, radiological characteristics, and the combination of both, respectively.

	Training cohort			Validation cohort		
	SVM1 (95% CI)	SVM2 (95% CI)	SVM3 (95% CI)	SVM1 (95% CI)	SVM2 (95% CI)	SVM3 (95% CI)
AUC	0.8170 (0.7353–0.8987)	0.91750 (0.86750–0.95850)	0.92350 (0.86460–0.98240)	0.80170 (0.68780–0.91570)	0.80880 (0.70640–0.91130)	0.90990 (0.83240–0.98730)
Specificity	0.86806 (0.79167–0.92361)	0.79861 (0.63889–0.94444)	0.916667 (0.861111–0.972222)	0.75806 (0.59677–0.90323)	0.70968 (0.46774–0.90323)	0.88710 (0.77419–0.98387)
Sensitivity	0.75610 (0.58537–0.87805)	0.92683 (0.73171–100.00)	0.878049 (0.756098–0.975610)	0.82353 (0.58824–100.00)	0.88235 (0.64706–100.00)	0.94118 (0.76471–100.00)
Accuracy	0.83784 (0.77297–0.89189)	0.82703 (0.71878–0.91892)	0.908108 (0.859459–0.951351)	0.78481 (0.65823–0.87383)	0.74684 (0.58228–0.86076)	0.89873 (0.81013–0.96203)
FNR	0.05405 (0.02703–0.09189)	0.01622 (0.000–0.05946)	0.027027 (0.005405–0.054054)	0.03797 (0.00000–0.08861)	0.22785 (0.07595–0.41772)	0.01266 (0.0000–0.05063)
FPR	0.10270 (0.05946–0.16216	0.15676 (0.04324–0.28108)	0.064865 (0.021622–0.108108)	0.18987 (0.07595–0.31646)	0.22785 (0.07595–0.41772)	0.08861 (0.01266–0.17722)

SVM, support vector machine; 95% CI, 95% confidence interval; AUC, area under the curve; FNR, false negative rate; FPR, false positive rate.

### Correlation Among All Features Used in Modeling

**Figure 5:**
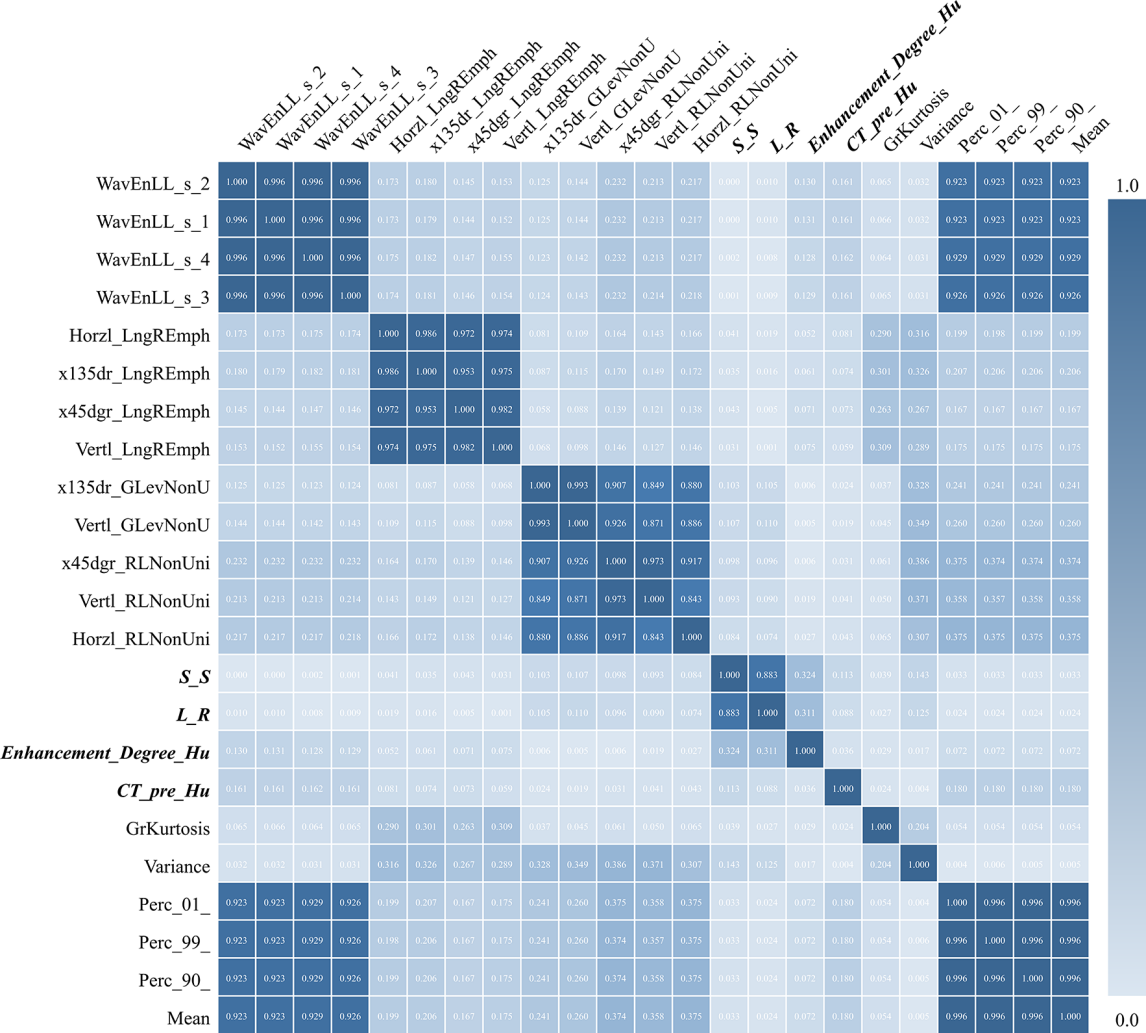
Correlation matrix test among all 19 radiomic textural features and four traditional radiological features (bold font) used in predictive modeling. S-S, indicating the craniocaudal dimension of the tumor; L-R, indicating the transverse dimension of the tumor.

We obtained a correlation matrix to evaluate the correlations among all the features included in the final model. As shown in **Figure 5**, the correlations were relatively high among the four selected traditional radiological characteristics (0.036–0.883), and were varied among the 19 selected radiomic textural features (0.000–1.000). Interestingly, although a few radiomic textural features (including Mean, Variance, Perc_01, and Perc_99) presented high correlation indices (0.923–0.929), the remaining 15 radiomic textural features had relatively low correlation indices (0.004–0.375), which justified using the features from both the radiomic texture and the traditional radiological assessment to build a more reliable predictive model.

## Discussion

In this study, we utilized pre-surgical CT images to develop a radiomic machine learning model for differentiating the low-grade from the high-grade ccRCC. Our machine learning models incorporating optimal radiomic textural features achieved an AUC up to 0. 92 in the training cohort and 0.91 in the validation cohort. Our study provided promising data for potentially using noninvasive imaging-based method to predict pathological grade of ccRCC.

We included several traditional radiological characteristics in the modeling process, including tumor size measurements, T staging information, and CT density values (21). These commonly used radiological characteristics have been used to predict tumor progression and pathological grade with some success (17, 21, 23, 24). However, to the best of our knowledge, our results showed for the first time that the model built with these traditional radiological characteristics was not stable enough to make a reliable prediction of pathological grade for ccRCC. Nevertheless, these radiological characteristics were visible to the human eye and could be conveniently assessed by radiologists and trained imaging personnel. These radiological characteristics have been valuable in clinical practice and we therefore should include them in predictive modeling. On the other hand, our study also showed low correlation index between the traditional radiological characteristics and the radiomic textural features, indicating these two different kinds of features may contribute different rather than redundant tumor information. Our study showed the potential of combining the observed radiological characteristics and the radiomic computational approach to improve model performance.

The mechanism underlying our satisfying radiomic model performance is not clear. Imaging features of tumor heterogeneity may represent the phenotypes of tumor (26, 33, 34). Tumor heterogeneity may potentially be expressed phenotypically in images as intratumoral heterogeneity and could be comprehensively assessed by imaging analysis (17, 33). Therefore, it is reasonable to speculate that radiomic textural features in our study may represent tumor heterogeneity, thus being relevant in predicting pathological grade as indicated in prior literature (26, 33–37). For texture features, we found that the features prompting the model to classify renal tumors as high-grade ccRCC mainly belonged to histogram (such as: variance), run-length matrix (such as: run length nonuniformity, and gray level nonuniformity. with the higher values of these textural features, there were corresponding higher LASSO scores, indicating the higher risk of the tumor being classified as a higher-grade ccRCC. Regarding the traditional radiological features, the LASSO regressors included the tumor size measurements, CTpre density value and the enhanced degree of the tumor. It is understandable that the larger the tumor poses the greater risk of being high-grade because of greater tumor heterogeneity. In addition, higher-grade tumors may have worse pathological differentiation and tumor necrosis, which may lead to a lower degree of enhancement.

Our study was generally in line with prior reports of renal cancer assessed with machine learning radiomics (29, 38–41). Our model performance was comparable to the prior studies which had AUC values reaching 0.86~0.98 for predicting pathological grade of renal cancers. However, our method for radiomic analysis was different from others in that we extracted radiomic features from one representative pre-enhanced axial CT image containing the maximal cross-sectional tumor dimensions while others obtained radiomics from contrast-enhanced images on both CT and magnetic resonance imaging. There were several advantages in our novel approach. First, our method was feasible and could be readily adopted as non-enhanced images were routinely included in CT imaging of renal cancer. It is easier to acquire the non-enhanced images than the contrast-enhanced images, and the image quality for the non-enhanced images could be better controlled than the contrast-enhanced images. In addition, our method could be used in patients who could not have contrast-enhanced imaging due to either contrast allergy or abnormal renal function which is especially relevant in patients with renal cancers. Secondly, the contrast-enhanced images may vary depending on the distribution and amount of contrast agents in the tumor tissue, which could be affected by multiple variables such as the type of contrast agent used, the injection speed, the hemodynamic conditions of the patients, etc (32). Therefore, our approach of using non- enhanced images could alleviate the concerns stemming from the potential image variations due to contrast enhancement. Lastly, our single image strategy could be useful for our planned multicenter clinical trials because of its simplicity to use and its readiness to be standardized among all participating centers. Furthermore, the acceptable efficiency of our method using the single image at the maximal cross-sectional tumor level has been reported in our own publication (32).

It should be noted that our satisfying model performance in predicting the pathological grade of ccRCC could be partly related to the classification algorithm used in the present study. This algorithm was developed by our team, and has been successfully applied in our current and previous studies (32). The basic logic of this classification algorithm was to treat LASSO and RF as weak regressors in the whole algorithm, which respectively reflected the classification attributes of the research object. We then used the SVM algorithm to combine these two to finally achieve the purpose of enhancing the classification effect. In addition, the final regressed scores from this algorithm could be binarized for further prediction. Nevertheless, our modeling algorithm was far from being comprehensive. More work is needed to further improve the classification performance by continually optimizing and improving the structure of the data mining algorithms.

We recognized that there was an apparent contradiction in our feature selection for the final model building. The contradiction was that we used the non-enhanced CT images for radiomic feature extraction but included the degree of CT enhancement as part of the traditional radiological characteristics in the final model. We believe we could resolve this apparent contradiction with the following explanation. First, the degree of CT enhancement was one of the most important radiological characteristics assessed by radiologists. Due to limitations of human visual inspection, the traditional radiological characteristics are usually limited in number including only the tumor size measurement and CT densities on both pre- and post-contrast images as in our study. Therefore, it was important to include it in the model building in our attempt to keep the few commonly reported characteristics which reflects the current clinical radiological practice. Second, while the traditional radiological assessment could only provide descriptive information on tumor characteristics, radiomics could extract a multitude of computational quantitative imaging features about tumor heterogeneity not visible to human eyes. Therefore, these two approaches in our study with one using non-enhanced images and another using enhanced images were complimentary rather than contradictory to each other and the combination of both strengthened the model performance as shown in our study.

This study had several limitations. First, this was a retrospective study conducted at a single institution, and case selection bias seemed inevitably. In addition, although there were 264 patients with ccRCC included in our study, our sample size was still modest for a machine learning study given heterogeneous disease distribution. Second, our validation cohort used to test the model efficiency was from the same institution as the training cohort, therefore making it challenging to generalize our results to other institutions and other disease settings. Future large-scale independent prospective multicenter studies are needed to validate our results. Third, our study was focused on ccRCC which constituted most of the renal cancer. However, it was not sufficient for a complete survey of renal cancer since other renal cancer subtypes could have similar imaging features and therefore should be evaluated in future studies. Moreover, our study was limited in that an accurate imaging-pathological correlation for each patient could not be performed in this retrospective study, which could have been helpful to assess the underlying pathological basis of our model performance. Lastly, the CT images in this study were obtained in three different CT scanners, which may be variable in terms of imaging quality due to inherent differences among the scanners. This may in turn potentially affect the textural features and model performance.

In summary, we developed a radiomic machine learning model with the pre-surgical CT images, achieving a satisfying performance in differentiating the low-grade from the high-grade ccRCC. Our approach integrating the traditional radiological characteristics and the radiomic textural features improved the performance of our prediction models. Our study presented a potentially useful non-invasive imaging-focused method to predict the pathological grade of renal cancers prior to surgery, which should assist in clinical decision making for selecting cancer treatment strategies and for informing prognosis.

## Data Availability Statement

The original contributions presented in the study are included in the article/**Supplementary Material**, further inquiries can be directed to the corresponding authors.

## Ethics Statement

This single-center retrospective study was approved by Ethic Committee and Institutional Review Board in Xiangya Hospital of Central South University, P. R. China (IRB#2017121011), and the written informed consents were waived.

## Author Contributions

XY and LL conceived and designed the study. XY, FZ, LL, QXi, CL, and GL analyzed the data. XG, QXi, HY, ZL, CQ, CW, XY, QXu, CQ, ML, and GG contributed analysis tools. XY, XG, LL, CZ, and BC wrote the paper. All authors contributed to the article and approved the submitted version.

## Funding

This study was supported in part by a project funded by the China Postdoctoral Science Foundation (2018M632997), the Postdoctoral Science Foundation of Central South University (No.185705), the Natural Science Foundation of Hunan Province (2018JJ2641), the Shenghua Yuying talents program at Central South University, P.R. China and the National Natural Science Foundation of China (81902727).

## Conflict of Interest

The authors declare that the research was conducted in the absence of any commercial or financial relationships that could be construed as a potential conflict of interest.

## Abbreviations

ccRCC, clear cell renal cell carcinoma; HE, hematoxylin and eosin; IHC, immunohistochemistry; ISUP, International Society of Urological Pathology; LASSO, least absolute shrinkage and selection operator; pRCC, papillary renal cell carcinoma; RCC, renal cell carcinoma; RF, random forest; SVM, support vector machine; WHO, World Health Organization.
