# Supplementary material for: Computed Tomography Radiomics for Predicting Pathological Grade of Renal Cell Carcinoma
Source: Front Oncol. 2021 Jan 27;10:570396. doi: 10.3389/fonc.2020.570396 (PMC7873602; doi:10.3389/fonc.2020.570396)
Supplement: Supplementary file 1 [file DataSheet_1.docx]

**Supplement files**

**Equations**

***Equation 1***

Rad-score=(4.9129e-06)*Mean+(0.00021328)*Variance+(2.8838e-11)*Perc_01_+(5.1856e-08)*Perc_90_+(1.0491e-07)*Perc_99_+(-0.0010872)*Horzl_RLNonUni+(2.7338e-05)*Horzl_LngREmph+(0.00029282)*Vertl_RLNonUni+(0.00012787)*Vertl_GLevNonU+(-2.3385e-05)*Vertl_LngREmph+(-1.1768e-05)*x45dgr_RLNonUni+(9.347e-06)*x45dgr_LngREmph+(5.1203e-05)*x135dr_GLevNonU+(-2.4036e-05)*x135dr_LngREmph+(-6.1467e-05)*GrKurtosis+(-1.3319e-05)*WavEnLL_s_1+(-4.5884e-07)*WavEnLL_s_2+(-1.3424e-06)*WavEnLL_s_3+(-3.319e-07)*WavEnLL_s_4

***Equation 2***

Score4= (0.026875)*L_R+(0.026351)*S_S+(0.013963)*CT_pre_Hu+(-0.0014128)*Enhancement_Degree_Hu

**Supplementary Table 1** Fuhrman and WHO/ISUP grading system

| Fuhrman grading system | | | |
| --- | --- | --- | --- |
|  | Nucleoli | Nuclear shape | Nuclear size |
| Grade 1 | absent or Inconspicuous | Small, round, uniform, Irregular | ~10μM |
| Grade 2 | Visible at x 400 magnification | Irregular in outline at x 400 magnification, variable in size | ~15μM |
| Grade 3 | Prominent/large even at x 100 magnification | Obvious irregular outline, large, hyperchromasia, marked variability in size and shape | ~20μM |
| Grade 4 | Cells large, pleomorphic with bizarre multilobed giant cells and heavy chromatic clumps, extreme irregular outlines | | |

**Supplementary Table 2** WHO/ISUP grading system

| WHO/ISUP grading system | |
| --- | --- |
| Grade 1 | Nucleoli are absent or inconspicuous and basophilic at x 400 magnification |
| Grade 2 | Nucleoli are conspicuous and eosinophilic at x 400 magnification and visible but not prominent at x 100 magnification |
| Grade 3 | Nucleoli are conspicuous and eosinophilic at x 100 magnification |
| Grade 4 | There is extreme nuclear pleomorphism and/or sarcomatoid and/or rhabdoid differentiation and/or multinucleate tumor giant cells |

| **Supplementary Table 3** Distribution of CT scans obtained in the 3 different scanners | | | | |
| --- | --- | --- | --- | --- |
|  | CT room1 | CT room2 | CT room3 | P value |
| ***ISUP grade (n, %)*** |  |  |  | 0.197 |
| Low grade | 110 (53.4) | 66 (32.0) | 30 (14.6) |  |
| High grade | 30 (51.7) | 24 (41.4) | 4 (6.9) |  |
| ***Cohort (n, %)*** |  |  |  | 0.983 |
| Training | 105 (52.2) | 67 (34.0) | 25 (12.7) |  |
| Validation | 140 (53.0) | 90 (34.1) | 34 (12.9) |  |

**Supplementary figure 1** The data mining algorithm, including data reduction process used in this study.

**
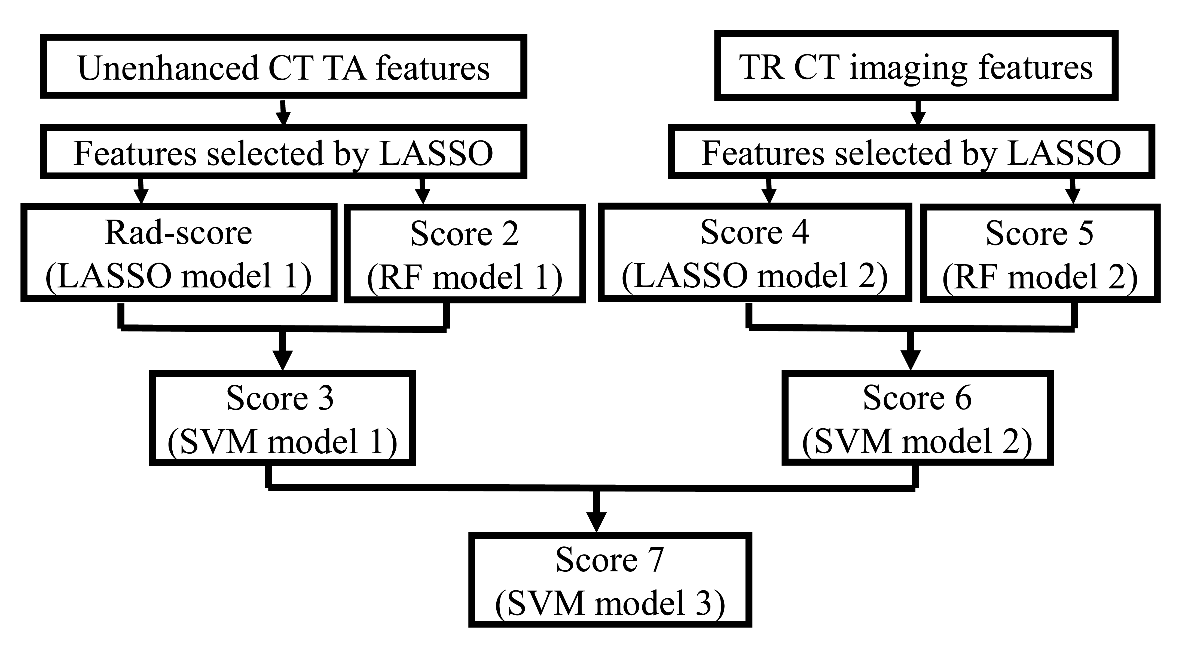
**
